# Cerebellar contributions across behavioural timescales: a review from the perspective of cerebro-cerebellar interactions

**DOI:** 10.3389/fnsys.2023.1211530

**Published:** 2023-09-07

**Authors:** Ellen Boven, Nadia L. Cerminara

**Affiliations:** ^1^Sensory and Motor Systems Group, Faculty of Life Sciences, School of Physiology, Pharmacology and Neuroscience, University of Bristol, Bristol, United Kingdom; ^2^Neural and Machine Learning Group, Bristol Computational Neuroscience Unit, Intelligent Systems Labs, School of Engineering Mathematics and Technology, Faculty of Engineering, University of Bristol, Bristol, United Kingdom

**Keywords:** cerebellum, supra-second timing, computational neuroscience, cerebral cortex, cerebro-cerebellar

## Abstract

Performing successful adaptive behaviour relies on our ability to process a wide range of temporal intervals with certain precision. Studies on the role of the cerebellum in temporal information processing have adopted the dogma that the cerebellum is involved in sub-second processing. However, emerging evidence shows that the cerebellum might be involved in suprasecond temporal processing as well. Here we review the reciprocal loops between cerebellum and cerebral cortex and provide a theoretical account of cerebro-cerebellar interactions with a focus on how cerebellar output can modulate cerebral processing during learning of complex sequences. Finally, we propose that while the ability of the cerebellum to support millisecond timescales might be intrinsic to cerebellar circuitry, the ability to support supra-second timescales might result from cerebellar interactions with other brain regions, such as the prefrontal cortex.

## 1. Introduction

Timing is crucial for wide-ranging behaviours: from our ability to catch a ball, cross a road, perceive music or plan our commute to work. Therefore, the ability to encode temporal information across a wide range of time scales is essential for generating adaptive behaviour that is key to our survival. Our capacity to behave adaptively results from our ability to learn by interacting with an environment in which states dynamically evolve across different timescales, ranging from slowly changing contextual states of the world to fast trajectories of bodily movement (Hasson et al., 2008; Kiebel et al., 2008; Chen et al., 2015). While it is known that neural circuits process temporal intervals during behaviour (Carr, 1993), how temporal information processing in the brain enables adaptive goal-directed behaviour remains unclear.

Many cerebellar studies have investigated cerebellar timing related to brief moment to moments such as limb and eye movements, whisking and finger tapping (e.g., Marple-Horvat and Stein, 1987; Ivry et al., 1988; Coltz et al., 1999; Thier et al., 2000; Medina and Lisberger, 2009; Chen et al., 2016; Nashef et al., 2018; Becker and Person, 2019; Cerminara et al., 2022). However, many behaviours require long-term planning, adaptation, attention and working memory, and as such, the cerebellum must play a role in timing on longer time scales (Popa et al., 2017), most likely through connections with higher brain regions in the cerebral cortex. One of the most prominent loops in the brain, that has expanded across evolution, is between the cerebrum and cerebellum (Rilling and Insel, 1998; Sultan, 2002; Herculano-Houzel, 2010). Despite growing evidence that the cerebellum forms reciprocal functional and anatomical loops with sensory, motor and associative cerebral areas (Allen and Tsukahara, 1974; Strick et al., 2009; Li and Mrsic-Flogel, 2020; Pisano et al., 2021; McAfee et al., 2022), the function of these biological feedback loops remains largely unknown in relation to temporal information processing. This review will provide a theoretical account of cerebro-cerebellar interactions with a focus on how cerebellar output can modulate cerebral processing during learning of complex sequences, and the extent to which the cerebellum is necessary for temporal processing supported by the cerebral cortex when perceiving time intervals in the supra-second time range, in other words seconds to minutes.

## 2. Evidence to suggest that the cerebellum contributes to information processing in longer time scales

**FIGURE 1:**
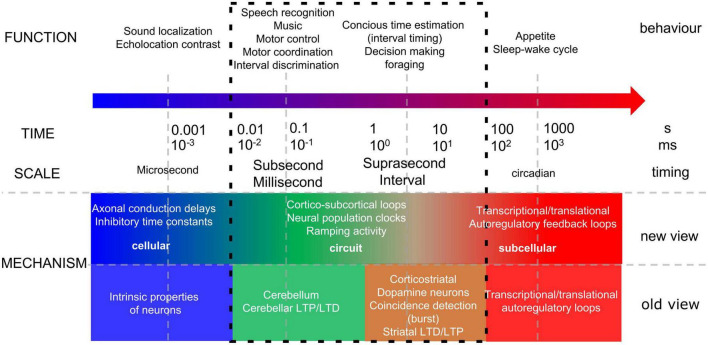
Temporal information processing. Schematic representing the complexity of behaviour, brain regions (old, Buhusi and Meck, 2005) and brain-wide feedback loops (new, Zhou and Buonomano, 2022) that underlie different scales of temporal information processing. Looking at the biological mechanisms underlying sub and supra-second timing, this used to be attributed to two distinct circuits (old view), but recent experimental evidence points out that the apparent division between sub- and supra-second timing systems (see box, new view) might not be as clear cut (see main text).

Our current understanding of the underlying neurobiological bases of temporal information processing, broadly speaking, involves two main circuits (see Figure 1, old view; Buhusi and Meck, 2005). On the one hand it is thought that the cerebellum is important for tracking the duration between events in the range of milliseconds, or sub-second timing. Indeed, the cerebellum is classically linked to a range of sensorimotor skills that require precise millisecond timing of the motor response (Timmann et al., 1999; Koekkoek et al., 2003; Lewis and Miall, 2003; Ivry and Spencer, 2004). Numerous studies in animals and humans have shown that the cerebellum is required for sub-second timing tasks such as finger tapping, eye blink conditioning and temporal discrimination tasks (e.g., Ivry and Keele, 1989; Garcia and Mauk, 1998; Medina and Mauk, 2000; Spencer and Ivry, 2013; Johansson et al., 2014). On the other hand, the basal ganglia and cerebral circuits, are thought to be required for the perception of more slowly evolving events, at the scale of seconds to minutes which guides adaptive behaviours such as foraging and decision making. Neural substrates underlying interval timing include, amongst others, thalamo-cortical-striatal circuits, with cortical regions including the prefrontal cortex (PFC) and the posterior parietal cortex (for review Buhusi and Meck, 2005).

More recent studies, however, have provided evidence for cerebellar involvement in the supra-second timing range (Nichelli et al., 1996; Mangels et al., 1998; Tracy et al., 2000; Gooch et al., 2010). For example, Gooch et al. (2010) observed overestimation as well as underestimation of supra-second time intervals in cerebellar patients with damage to the lateral areas of the cerebellum. In addition, studies have observed co-activation of the cerebellum with the prefrontal cortex during supra-second timing tasks and that stimulation of cerebellar projections to prefrontal cortex can rescue timing deficits in schizophrenia (Parker, 2016; Parker et al., 2017; Heskje et al., 2020). Whereas in these studies timing can be regarded as explicit, in many decision-making and working memory tasks, timing is implicit as it involves prediction of events (Bares et al., 2019). However, the current state of research suggests a broader role for the cerebellum in event timing and thus appears independent of such a dichotomy (Ivry et al., 2002; Breska and Ivry, 2016; Bares et al., 2019; Gaffield et al., 2022), with the cerebellum also playing a role in processing longer timescales. First, several studies have shown that Purkinje cell simple spike discharge includes short- and long-range representations of both upcoming and preceding behaviour (Popa et al., 2017, 2019). Another study in zebrafish observed how Purkinje cells use prediction errors to acquire an internal model of supra-second stimulus timing (Narayanan et al., 2021). Such physiological mechanisms could underlie cerebellar involvement in error correction, working memory and sequencing over longer time intervals (Deverett et al., 2018; Rondi-Reig et al., 2022), particularly through its connections with the cerebral cortex. Together, these reports question the dogma that the cerebellum exclusively influences sub-second timing (Ohmae et al., 2017), indicating that the apparent division between sub- and supra-second timing systems might not be as clear cut (see Figure 1, new view, Petter et al., 2016; Zhou and Buonomano, 2022). However, a unified perspective of the physiology and anatomy of the cerebellar involvement in supra-second timing is currently lacking. We propose that cerebellar contributions to supra-second timing arise from its interactions with the cerebral cortex.

## 3. Cerebro-cerebellar interactions

The cerebellum is bidirectionally connected to the cerebrum via cerebro-cerebellar circuits (Apps and Watson, 2013). In one direction, information descends from different parts of the cerebral cortex toward the cerebellum, via several structures in the brainstem. Several studies highlight the impact of a range of cerebral areas (sensory, motor and association areas) onto the cerebellum via the mossy fibres coming from the pons (Legg et al., 1989; Odeh et al., 2005) to form the cerebro-ponto-cerebellar projection (Armstrong, 2022). Besides the pontine mossy fibre system, cerebro-cerebellar communication is also mediated by the climbing fibre system, via the cerebro-olivo-cerebellar pathway, via projections through the mesodiencephalic junction. For example, one study using *trans*-neuronal tracing has revealed that connections from the cerebral cortical areas onto different olivary subnuclei are topographically organised (Wang et al., 2022). Together, the mossy fibre and climbing fibre systems give the cerebellum access to the temporal hierarchy of information that exist across the neocortex (Hasson et al., 2008; Murray et al., 2014) to integrate and utilise such information to relay to downstream areas or back to the cerebral cortex.

In the other direction, output from the cerebellar nuclei projects via the thalamus to the cerebrum (Habas et al., 2019). The traditional view is that cerebello-thalamic projections arising from the three output nuclei project primarily to the motor cortex (Nashef et al., 2019, 2021). However accumulating evidence shows that the cerebellar paths to the cerebral cortex are more distributed. For example, the enlarged size of the human dentate nucleus, a cerebellar output channel which projects to non-motor cerebral areas (Dum and Strick, 2003), provides evolutionary evidence for the proposed role of the cerebellum in higher order thinking (Leiner et al., 1993). Cerebellar pathways to frontal areas of the cerebral cortex have now also been identified in rodent models, with the principal route of cerebellar feedback projections to the cerebrum occurring via the ventral thalamic nuclei (Pisano et al., 2021). Although other, indirect, routes via subcortical and brainstem structures such as basal ganglia and tegmental areas are possible, the cerebello-thalamic projections are rapidly conducting, thus providing a substrate for reciprocal communication between cerebellum and the cerebral cortex (Watson et al., 2009, 2014). This global input and output diversity, together with local diversity (see De Zeeuw et al., 2021), of the modular architecture thus enable the cerebellum to support a wide range of behaviourally relevant temporal contingencies, not only the fast-fluctuating patterns related to sensory motor control.

Several studies on how the neural dynamics of cerebral cortex and cerebellum depend on each other have supported the notion that the cerebellum contributes to higher order processing via cerebro-cerebellar interactions. Evidence from *in vivo* recordings and theoretical studies have indicated that diverse neural representations can be faithfully transmitted between the cerebral cortex and cerebellum via the intermediate structures such as the pons and thalamus (Wagner et al., 2019), which are thought to enable optimal transformation of electrical activity between the brain areas (Lakshminarasimhan et al., 2022; Muscinelli et al., 2022). Additionally, studies focussed on how the cerebellar output influences cerebral activity, implicate the cerebellum as a driver of cerebral activity dynamics underlying goal-directed behaviour (Dacre et al., 2021). Using optogenetics combined with *in vivo* electrophysiology, Gao et al. (2018) found that cerebellum is required to maintain preparatory motor activity in the premotor cortex that are necessary for task execution. Another study contributed to this idea by showing that the cerebellum learns to predict the timing of upcoming rewards to sculpt the preparatory representations in the cerebral cortex (Chabrol et al., 2019). From a theoretical perspective the idea of the cerebellum as a driver of cerebral dynamics seems plausible given the characteristic computational features that arise from the contrasting circuit architectures.

## 4. Cerebro-cerebellar interactions for temporal information processing

From a theoretical perspective the problem of learning what happens when, is known as the temporal credit assignment: the process of identifying which set of past actions and observations, and their underlying neural representations, lead to a favourable behavioural outcome (Sutton, 1984). Recurrent neural networks—brain-inspired artificial architectures—are characterised by reciprocal connections providing inherent feedback loops of information and have been shown to process time-dependent sequences (Hardy and Buonomano, 2018). This is in stark contrast to a feedforward network, in which processing depends on current inputs without fluctuations of previous inputs propagating over time (Elman, 1990). As a result of their ability to encode temporally varying sequences, recurrent neural networks have been at the forefront as computational models for the neural basis of temporal information processing, and are widely used in computational neuroscience to model behaviourally relevant sequences (Murugan, 2018; Kaushik et al., 2023).

**FIGURE 2:**
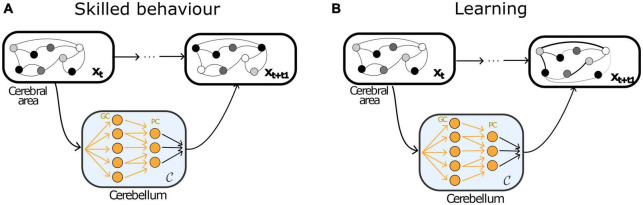
Cerebro-cerebellar loop as artificial neural networks. Schematic representation of the cerebro-cerebellar loop using artificial neural networks. While the cerebrum is characterised by mainly recurrent local connectivity, the cerebellum can be approximated by a feedforward neural network. **(A)** During skilled behaviour, the cerebellum uses the current cerebral state to predict the next. **(B)** In contrast, there is currently no computational account of how the cerebellum interacts with the cerebral cortex during learning that involves the acquisition of appropriate cerebral activity patterns through changes in connectivity. Image adapted from Tanaka et al. (2020).

Artificial recurrent neural network models have been successful at approximating the cerebrum as a dynamical system (Laje and Buonomano, 2013; Mante et al., 2013; Rajan et al., 2016; Song et al., 2016). The recurrent connections allow for input information to be sustained and propagated over time, whereas processing in a feedforward network only depends on current inputs, so the encoded information depends less on information carried over from previous events. A recurrent neural network is also known to be difficult to train and control because it may exhibit chaotic behaviour (Sompolinsky et al., 1988; Sussillo and Abbott, 2009; Laje and Buonomano, 2013). A feedforward neural network, on the other hand, is stable because its output depends not on previous inputs but only current inputs and a fluctuation at one point of time does not propagate over time. Previous theoretical studies have shown that stable activity patterns in recurrent neural network can be generated by adding a non-recurrent feedback connection from the output to the recurrent units (Sussillo and Abbott, 2009; Ben-Shushan and Tsodyks, 2017). Taken together, the experimental and theoretical evidence support the idea that the cerebellum drives cerebral dynamics by predicting the next state based on the copy of cerebral dynamics it receives. Based on this, Tanaka et al. (2020) propose that the computational role of the cerebellar output to the neocortex is to stabilise recurrent cerebral dynamics by predicting the expected activity of the cerebral cortex (Figure 2A).

Another study implicates the cerebellum in shaping motor commands in the motor cortex by conditioning cerebral plasticity using predictions of sensory feedback (Popa et al., 2013). The idea that the cerebellum learns internal models of sensory feedback together with its modular organisation support this idea. Moreover, the existence of non-uniform cerebellar modules (Cerminara and Apps, 2011; Ruigrok, 2011; Apps et al., 2018), composed of topographic-specific global connections and local diversity in cerebellar circuitry, suggest that the cerebellum can facilitate tuning of task-specific representations by simulating feedback across a range of modalities, including physical and internal states.

To date, there has been little to no theoretical work that postulate constraints on how the cerebellum interacts with the cerebral cortex during acquisition of skilled behaviour. One attractive framework comes from the idea that associative learning in cortical networks can approximate the back-propagation algorithm (Whittington and Bogacz, 2019). Together, two gaps in the understanding of cerebro-cerebellar networks can be identified. First, most studies looking at cerebro-cerebellar interactions have focussed on tasks that engage cerebellar connections with motor and premotor cerebral areas, while studying the role of the cerebellum in tasks that engage other cerebral areas remain unexplored. And second, is the idea that the cerebellum exerts a, potentially complementary (Stein, 2021) role on cerebral areas during learning, in which the cerebellum facilitates acquisition of appropriate activity patterns through changes in cerebral connectivity. Such an interaction is currently unaccounted for in computational models of these circuits (Figure 2B).

## 5. Models of cerebro-cerebellar circuits

Recently, a computational model of cerebro-cerebellar interactions for temporal credit assignment was developed. Boven et al. (2023) suggested that the main function of the cerebellar circuit during learning is to provide a feedback signal that enables the cerebral cortex to acquire adaptive representations by increasing the amount of temporal information available to each cerebral network. More specifically, a cerebellar, feedforward, network communicates with a cerebral, recurrent network forming a two-learner system in which the cerebellar network effectively learns to provide internal feedback signals to the cerebral network. The main network then integrates these internal feedback signals. The cerebellar signal, which contains information about future feedback that the cerebral cortex receives, influences the cerebral network such that appropriate activity patterns can be acquired for precise behaviour. In the model presented the cerebellar feedforward network is tasked with predicting future feedback giving the current state of the cerebral RNN. The amount of temporal information refers to task-specific information by making feedback available beyond the intrinsic scale of the cerebral network. The cerebellum thus learns to predict this feedback based on the neural representation in the cerebral cortex, thereby decoupling learning in cerebral networks from future feedback.

**FIGURE 3:**
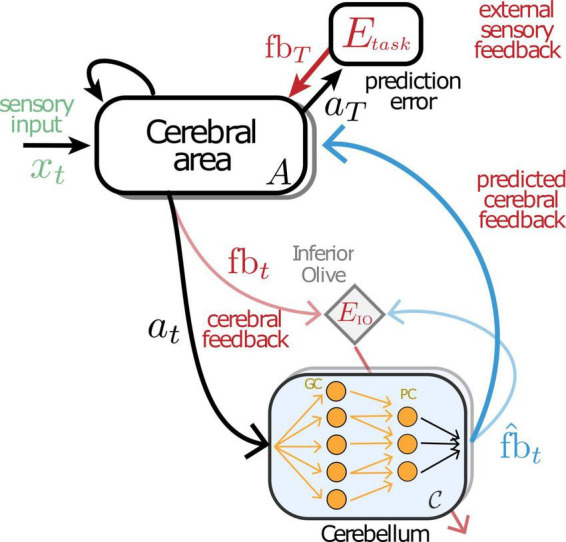
Schematic representation of the cerebro-cerebellar model. A cerebellar feedforward network is connected to a recurrent cerebral module. The cerebellum continuously predicts the feedback expected by the cerebral network *fb*_*t*_ (blue) given current cerebral activity *a*_*t*_ (black). The cerebellar network, consisting of granule cells (GC) and Purkinje cells (PC), learns through prediction errors (bottom red arrow) computed at the inferior olive (diamond) by comparing predicted cerebral feedback *fb*_*t*_ with actual cerebral feedback *fb*_*t*_ (light blue). Image adapted from Boven et al. (2023).

The cerebro-cerebellar model (see Figure 3) suggests that the cerebellum mediates behaviour by predicting feedback across a range of time scales. When trained in sensorimotor tasks the model shows faster learning and reduced dysmetria-like behaviours, in line with normal cerebellar function. These results indicate that cerebellar feedback predictions enable the cerebral cortex to acquire adaptive representations effectively by increasing the amount of temporal information available to each cerebral network. The cerebellar feedback signals facilitated learning especially when there was a limited amount of feedback information coming from the environment or internal body state. This is highly relevant for reward-based learning. In addition, the authors show that the cerebro-cerebellar model is applicable to a wider range of cognitive tasks that evolve over longer timescales, while being inspired by a body of work showing language deficits in cerebellar patients (e.g., Stoodley and Schmahmann, 2009; Guell et al., 2015). More specifically, the model was implemented in a recreating sentence task studied by Guell et al. (2015) in cerebellar patients, which showed a poor semantic description of images when compared to healthy subjects. To demonstrate a similar behaviour in the model, the authors built on existing datasets commonly used in caption generation tasks in machine learning. In this task the model is provided with a low dimensional representation of a natural image and the RNNs are then trained to consequently predict the next word given the compressed sensory input and the previous word. The results show that the cerebellar enhanced model generates richer captions and that it is particularly beneficial for longer captions, suggesting that the cerebro-cerebellar model can learn richer visuo-language contextual information. Specifically, the cerebellar feedback signal increased the amount of temporal information available to the cerebral network during learning and as such facilitated the acquisition of efficient task-relevant representations.

The model predicts that the cerebellum is particularly important for temporally challenging tasks, offering a potential explanation for recent experimental observations (Locke et al., 2018). This is because the cerebro-cerebellar model enables efficient temporal credit assignment. Therefore, this work suggests that the cerebellum reduces the need for strong temporal credit assignment in the brain. This predicts that when the cerebellum is perturbed the cerebrum must encode and learn with richer temporal signals to achieve a similar performance when compared with healthy controls. Moreover, the cerebellum has long been known to be involved in timing prediction (Ivry et al., 2002; O’Reilly et al., 2008). The model is related to these observations in that the cerebellar module learns to predict cerebral feedback at specific points in time, thereby providing moment-to-moment precision within the encoding of longer timescales. Although in the model these predictions are used directly for learning, it is possible that these temporal predictions have a broader impact on network dynamics and information processing in the brain, thus taking more the role of a driver than modulator (Pemberton et al., 2022).

Whereas the above computational work proposes a role for how the cerebellum affects cerebral areas during learning across sub- and supra-second timescales, recent experimental work shows that pharmacological inactivation of the lateral cerebellum does not impair supra-second peak-interval timing tasks in rats (Heslin et al., 2022). Using a nose-poke task to earn a water reward, the rats were trained to estimate a target interval. Heslin et al. (2022) tested the rats’ ability to learn a new supra-second target duration by introducing a longer interval once the animals had already been trained on a shorter interval. No deficit was observed in the animals to estimate the new target interval. One could argue that while the computational model by Boven et al. (2023) tests the acquisition of a novel task, the experimental work by Heslin et al. (2022) tests the generalisation of an already learned behaviour to a novel interval. Additionally, a requirement of the peak interval task is that the rate of nose pokes peaks around the target interval time. However, the nose poke can start a certain time before the target time. As the main requirement of the peak interval task is that the rate of nose pokes peaks around the target interval time, the studied behaviour does not require a well-timed movement. Additionally, the animals were not restricted from performing extraneous movements such as grooming. The use of stereotyped behaviours such as grooming in rodents has been shown to contribute to the ability to facilitate timing accuracy (Gouvea et al., 2014; Safaie et al., 2020). This suggests that the absence of impairment in the task by Heslin et al. (2022) could be due to stereotyped behaviours being performed during the target interval, or because the nose poke movement was not stereotyped and therefore not cerebellar dependent. Moreover, research in humans has identified differences in projections coming from dorsal vs. ventral dentate nucleus, with the latter being implicated in cognitive behaviours (Shipman and Green, 2020). It remains to be determined if such projections exist in the rodent cerebellum. This poses interesting questions for the requirements of how the cerebellum interacts with the cerebral cortex during the process of integrating temporal, structural knowledge, with skilled goal-directed movements.

While the study by Boven et al. (2023) suggests that the cerebellum is critical for learning temporal tasks on a supra-second timescale, future studies are required to understand the neural circuit mechanisms by which the cerebellum supports encoding of supra-second intervals. It is possible that, given its mainly feedforward structure, cerebellar computation for behaviour over a longer time scale could be broken down into sub-second time scales. Indeed, given the cerebellum’s feedforward structure and intricate connectivity with the rest of the brain it is likely that it makes predictions, and provides feedback, over multiple time horizons: from milliseconds (motor control) to seconds or more (decision-making, long-term motor planning). While its ability to support millisecond timescales might be intrinsic to cerebellar circuitry, the ability to support supra-second timescales might result from cerebellar interactions with other brain regions, such as the prefrontal cortex (Kim et al., 2009; Xu et al., 2014; Yin et al., 2019; Meirhaeghe et al., 2021; but see Caro-Martín et al., 2015). Additionally, connections between the cerebellum and basal ganglia have been well-characterised providing an additional substrate in timing that has not been considered in this review (Bostan et al., 2013; Petter et al., 2016). But as a first step toward a mechanistic understanding of cerebellar contributions to interval timing, it is useful to consider a computational model of cerebro-cerebellar circuits like the one presented by Boven et al. (2023) or variants thereof (Tanaka et al., 2020; Pemberton et al., 2022). The overarching idea being that the cerebral recurrent neural network is connected to a cerebellar network which receives a copy of the recurrent neural network activity and returns a prediction of the desired task outcome or feedback signals necessary for learning. Thus, future experimental studies could consider such a network architecture while implementing timing tasks.

### 5.1. Permission to reuse and copyright

Figures, tables, and images will be published under a Creative Commons CC-BY licence and permission must be obtained for use of copyrighted material from other sources (including republished/adapted/modified/partial figures and images from the internet). It is the responsibility of the authors to acquire the licences, to follow any citation instructions requested by third-party rights holders, and cover any supplementary charges.

## Author contributions

EB wrote the first draft of the manuscript. NLC contributed to writing and editing. Both authors contributed to the development of the concept.
